# Brain CT-Scan Findings in Unconscious Patients after Poisoning

**Published:** 2011-03

**Authors:** Morteza Sanei Taheri, Maryam Noori, Majid Shakiba, Amir Hossein Jalali

**Affiliations:** 1 *Department of Radiology, Loghman Hakim Hospital, Shahid Beheshti University of medical Sciences, Tehran, Iran;*; 2 *Department of Radiology, Loghman Hakim Hospital, Shahid Beheshti University of medical Sciences, Tehran, Iran;*; 3 *Research unit, Medical Imaging Center, Imam Khomeini Hospital Tehran University of medical Sciences, Tehran, Iran*

**Keywords:** poisoning, computed tomography, brain, neuroimaging

## Abstract

The aim of this study was to identify and describe brain CT findings in patients with poisoning or drug overdose and altered mental status. In this study, 403 patients with some degree of loss of consciousness who referred due to poisoning or drug overdose were evaluated by brain CT. The most common cause of intoxication was suicide. Intoxication status was determined by the physician and was mainly based on a history of intoxication, positive toxicologic screen result, or physical evidence suggesting intoxication. Among 403 unconscious patients, 229 patients who were ingested or inhaled Benzodiazepine, Carbamazepine, Carbon Monoxide, Ethanol, Methanol, Opium, Tricyclic antidepressants, and Tramadol included in the study. Others had used multiple drugs and/or toxins, or their intoxication was unknown. Mean age of patients was 37.6 ± 17.7 years (14-95). Among them, 181 (79%) were male. Among all patients, 92 had consumed opium (40.2%), 47 had consumed Benzodiazepines (20.5%) and other patients had been overdosed by other drugs or exposed to other poisonous agents. Totally 38 (16.5%) patients had abnormal CT findings. These included 10 cases of infarction, four cases of hemorrhage, two cases of herniation, 13 cases of edema, and 10 cases of basal ganglia changes (including 9 cases of hypodensity and one case of hypodensity with hemorrhage). A good knowledge of the CT findings in unconscious patients due to poisoning or drug overdose seems to be necessary for radiologists and clinicians. This study is unique in that it reported most of the radiological findings in these patients.

## INTRODUCTION

Poisoning is the third cause of injury related deaths in the United States (1).

The incidence of intoxication is about 1470 per 100/000 in the united stated, with 2.4 million exposures per year (2).

In poisoned patients who refer to the emergency department with unconsciousness, the importance of early radiologic evaluation has been recognized and accepted. CT-scan has greatly facilitated the early diagnosis of potentially life – threatening intracranial pathologies (3).

In patients who are known cases of poisoning, CT-scan can be useful in evaluation of the presence of toxin or its effects on the patient.

CT-scan is able to demonstrate toxic effects of methanol and many other toxins in the central nervous system.

In other cases that present with unconsciousness and the cause of this situation is unclear CT-scan may be useful in the diagnosis of intoxication.

Brain atrophy, intracranial hemorrhage, cerebral edema, ischemia, and infarction are some sequels of toxins in CNS and recognition of a toxin as the main cause of these conditions is critical in preventing mortality or further morbidity in these patients (4).

In previous studies putaminal necrosis, cerebellar and hypothalamic focal lesions, sub cortical white matter demyelization, and intracranial hemorrhage have been described in patients with methanol intoxication (5, 6).

Also loss of brain substance and subsequent brain atrophy has been identified in CT-scan of poisoned patients with psychoactive substances such as alcohol, benzodiazepines, heroin, and cannabis (7-10).

The aim of this study was to report abnormal brain computed tomography (CT) findings in a large group of patients with poisoning or drug overdose associated with altered mental status.

## PATIENTS AND METHODS

We performed this cross sectional study on 403 consecutive unconscious patients with suspected poisoning or drug over dose who who presented to the Loghman-Hakim Poison Hospital (LHPH) in Tehran, Iran were evaluated. The LHPH serves as a tertiary referral center for a population excess of 12 million in the capital of the country and normally sees 28,000 emergency ward presentations due to poisoning and about 12,500 of them are hospitalized each year. It seems that this complex to be the biggest clinical toxicology department in the world from December 2005 to December 2006.

This study was approved by our university affiliated hospital’s ethics committee on clinical investigations.

We included patients with presentations that appeared to be related to over dose or poisoning according to standard poison control center criteria (11).

These standard criteria were the patient's relative report of ingestion, a witnessed of drug ingestion, finding of empty drug boxes, or a suicide note combined with presentations related to a drug over-dose or poisoning, and toxicology testing when available.

All patients showed some degree of unconsciousness and underwent brain CT-scan during the first 24 hours of admission, on a Shimadzu (7800-JAPAN) system.

All CT-scans had been performed with the same scanner using the sequential techniques (collimation 10 mm, spacing 10 mm).

The window widths ranged from 100 to 120 haunsfield unit (HU) and levels ranged from 35 to 49. In the presence of head trauma history, bone window was performed with window widths of 2000 and level 400, then radiologic features were determined.

Brain swelling was classified as three categories: Mild, when the cortical sulci, Sylvian fissure, third ventricle and perimesencephalic cistern were compressed, but visible, in CT. Moderate, when one or two of these structures were not visible. Severe, when three or four of above structures were not visible.

Statistical analyses were done by SPSS ver 11.5.

## RESULTS

Among 403 unconscious patients, 229 who were ingested, or inhaled Benzodiazepine, Carbamazepine, Carbon Monoxide, Ethanol, Methanol, opium, Tricyclic antidepressants, and Tramadol included in the study. Others had used multiple drugs and/or toxins, or their intoxication was unknown.

Mean age of patients were 37.6 ± 17.7 years old (14-95). Among them, 181 (79%) were male and 48 (21%) were female. Among all patients, 92 had consumed opium (40.2%), 47 had consumed benzodiazepines (20.5%) and other patients had been overdosed by other drugs or exposed to other poisonous agents (Table 1).

**Table 1:** Distribution of drugs among patients

Type of Intoxication	Frequency	Percent

Benzodiazepine	47	14.9
Carbamazepin	15	4.8
CO	21	6.7
Ethanol	10	3.2
Methanol	12	3.8
opium	92	29.2
TCA	16	5.1
Tramadol	16	5.1

Among these 229 patients; only 38 cases (16.5%) had abnormal CT scan findings. Of these, four patients had skull fracture (three cases showed hemorrhage that could be referred to trauma). Remained 34 patients (14.8%) had abnormal CT scan findings unrelated to trauma. These included 10 cases of infarction, four cases of hemorrhage, two cases of herniation, 13 cases of edema, and 10 cases of basal ganglia changes (including nine cases of hypodensity and two cases of hypodensity with hemorrhage).

### Benzodiazepine group

Totally 47 patients were seen in this group; of them 36 were male (76.6%) and 11 were female (23.4%). Mean age was 34.6 ± 18.4 years old (16-90). 44 cases had suicide (93.6%) and the others had misusing the drug. 12 cases presented with coma (25.5%) and the others had some degrees of loss of consciousness. Totally 5 cases had brain abnormal CT-scans (10.6%). The findings of patients included; one case of infarction (2.1%), two cases of edema (4.2%) , one case of edema with basal ganglia changes (2.1%), and one case of herniation with edema (2.1%).

### Carbamazepin group

Totally 15 patients were seen in this group. Among them, 10 patients were male (66.7%) and 5 were female (33.3%). Mean age was 28.7 ± 12.2 years old (14-50). 11 cases were hospitalized because of suicide (93.6%) and the others because of misusing the drug. 5 cases presented with coma (33.3%) and the others had some degrees of loss of consciousness. One case had brain abnormal CT-scans (8.5%). This patient had mild edema in the CT scanning.

### Carbon Monoxide group

Totally 21 patients were seen in this group; of them 12 were male (57.1%) and 9 were female (42.9%). Mean age was 37.9 ± 15 years old (22-70). One case were hospitalized because of suicide (4.8%), 2 because of misusing (9.5%) and the others for uncurious use (18, 85.7%). 11 cases presented with coma (52.45%) and the others had some degrees of loss of consciousness. Totally five cases had brain abnormal CT scans (23.8%). The findings of patients were included: three cases of edema (14.3%) [One mild and two moderate], and two cases of basal ganglia changes (9.5%) (Both of them had hypodensity).

### Ethanol

Totally 10 patients were seen in this group and all of them were male. Mean age was 32.2 ± 12.5 years old (17-50). 9 cases were hospitalized because of misusing (90%). Two cases presented with coma (20%) and the others had some degrees of loss of consciousness. Two cases had abnormal brain CT scans (20%) due to skull fracture that one of them was accompanied with intracranial hemorrhage.

### Methanol

Totally 12 patients were seen in this group and all of them were male. Mean age was 43.9 ± 20.3 years old (16-75). 8 cases were hospitalized because of misusing the drug (66.7%). 6 cases presented with coma (50%) and the others had some degrees of loss of consciousness. Totally eight cases (67%) had brain abnormal CT-scans. The findings of patients included: one case of skull fracture that was accompanied with intracranial hemorrhage, one case of intracranial hemorrhage without fracture, and six cases of basal ganglia changes (50%) [5 of them presented with hypodensity and the other one with both hypodensity and hemorrhage].

### Opium

Totally 92 patients were seen in this group. Among them 79 were male (85.9%) and 13 were female (14.1%). Mean age was 44.0 ± 18.6 years old (15-95). 11 cases were hospitalized because of suicide (12%) and 74 (80.4%) because of misusing the drug. 27 cases presented with coma (29.2%) and the others had some degrees of loss of consciousness. Totally 14 cases had brain abnormal CT scans (15.2%). The findings of patients included: seven cases of infarction (7.6%), two cases of hemorrhage (2.2%), three cases of edema (3.3%) [2 mild and one severe], one case of basal ganglia changes (1.1%) (hypodensity), and one case of infarction and hemorrhage(1.1%).

### Tricyclic antidepressants

Totally 16 patients were seen in this group; of them 10 were male (62.5%) and 6 were female (37.5%). Mean age was 29.3 ± 11.3 years old (18-62). 15 cases were hospitalized because of suicide (93.8%) and 1 (6.2%) because of misusing the drug. 6 cases presented with coma (37.5%) and the others had some degrees of loss of consciousness. Totally 3 cases had brain abnormal CT scans (18.8%). The findings of patients included: one case of infarction (6.3%), one case of edema caused uncal herniation (6.3%), and one case of edema (6.3%).

### Tramadol

Totally 16 patients were seen in this group; of them 12 were male (75%) and 4 were female (25%). Mean age was 24.8 ± 6.8 years old (16-42). All cases were hospitalized because of suicide. All cases presented with some degrees of loss of consciousness. One case had brain abnormal CT scans (6.3%). The findings of this patient were hemorrhage with hernia and midline shift that was accomplished by skull fracture (thus this hemorrhage could be due to head trauma with skull fracture).

## DISCUSSION

Altered mental status is common in patients with poisoning or drug over dose.

Typical presentations of drug overdose include decrease in consciousness, agitation, seizures, or movement disorder.

Mental disturbances may occur due to pharmacologic effects of the drug (functional) or may be resulted by trauma or a drug-induced vascular accident (structural) or combination of these (12).

The aim of brain CT-scan is rapid diagnosis causes of altered mental condition, such as traumatic intracranial hemorrhage or hematoma.

Focal cerebral ischemia of the basal ganglia is one delayed CT-scan finding reported after cyanide, methanol, and carbon monoxide poisoning (13-15).

Another delayed complication of poisons which occur 1 or two days after poisoning is brain edema (16, 17).

One probable risk for obtaining CT-scan in a poisoned patient is transporting the patient from emergency ward for CT-scan. Thus poisoning alone dose not seem to be an indication to obtain brain CT-scan.

We all know that MRI is more sensitive than CT for the detection of tissue edema, and also MRI shows poor sensitivity to calcification and it is usually difficult to detect delayed calcified granules. Indeed, MRI gives a better resolution for cortical lesions than CT and also gives better anatomical detail in the case of hemorrhage.

Herein we discussed about brain CT-scan findings in our unconscious patients.

### Methanol poisoning

Methanol is a clear, colorless, highly toxic with a weak odor, slightly sweeter than ethanol.

Methanol has a toxic effect on the central nervous system.

Bilateral basal ganglia and putaminal necrosis, hypothalamic and cerebellar focal lesions, subcortical white matter demyelination, and edema with hemorrhage in the temporal lobe and bilateral occipital lobes have been reported in patients with methanol poisoning (5, 6).

Putaminal lesions and peripheral white matter findings can also occur in Wilson's disease, (18) Leigh's disease, Kearns-sayre syndrome, and striatal degeneration related with leber’s optic atrophy (19, 20).

Hemorrhage occurs in 13.5% of patients with methanol poisoning that may be due to the direct effects of formate in CNS (21).

We found bilateral symmetrical hypodense lesions in the basal ganglia in 50% of cases with methanol poisoning.

One was associated with basal ganglia hemorrhage and Para sagittal ICH, also two patients was associated with white matter hypodensity.

The hemorrhage was occurred in one of our patients before he underwent hemodialysis, such as Patankar *et al*. patient (6). However, in another study, the hemorrhage in the temporal lobe and bilateral occipital lobes occurred after hemodialysis (5).

Finally we think that hemorrhage in is unrelated to heparinization during hemodialysis.

### Tricycle antidepressant overdosage

Tricycle antidepressants (TCA) are among the most common prescribed medications in the world.

Due to their wide availability and frequent usage, TCA overdose is frequent. In one study, fujino *et al*. reported a case of TCA intoxication that her CT-scan showed progressive cerebellar atrophy (22).

To the best of our knowledge there are no any other reports about CT-scan findings in these cases in the literature.

In our study two cases had brain edema, one mild grade and the other with severe cytotoxic brain edema (irreversible type).

### Carbon monoxide intoxication

Carbon monoxide (co) intoxication is an emergency condition, which can result in neurological defects or death.

Co intoxication may cause different structural defect in the brain.

The most common seen changes are bilateral ischemic lesions and necrosis in the grey matter, especially in the globus pallidus (23, 24).

Narcissi *et al*. reported necrotic lesions in the purkinje cells of cerebral cortex, in the dentate nucleus, and in the cortex after co intoxication (25).

In our study two cases with basal ganglions hypodensity similar to that reported by Yuksel *et al* (26).

### Opium intoxication

Opium is the most common substance that has been abused in our country.

Brain infarction, edema, hemorrhage, and hypodensity changes in basal ganglia were seen in our patients with opium intoxication.

Hypoxic brain injury, transverse myelitis, brain atrophy, and toxic leukoencephalopathy were reported as the neuroimaging features of Heroin intoxication (10, 27).

But there is no any report about the effects of opium intoxication.

In conclusion, a good knowledge of the CT-scan findings in unconscious patients due to poisoning or drug overdose seems to be necessary for radiologists and clinicians. This study may be useful in that it reported most of the radiological findings in these patients.
